# Development and validation of a national clinical pharmacy competency framework for hospital pharmacists in Austria: a multi-method study

**DOI:** 10.1007/s11096-024-01781-3

**Published:** 2024-08-07

**Authors:** J. T. Stoll, B. Böhmdorfer-McNair, M. Jeske, A. E. Weidmann

**Affiliations:** 1 Department of Clinical Pharmacy, Innsbruck University, Innrain 15, 6020 Innsbruck, Austria; 2 Hospital Pharmacy, Hietzing Clinic, Vienna Health Association, Wolkersbergenstraße 1, 1130 Vienna, Austria; 3 Karl Landsteiner Institute for Clinical Risk Management, 1130 Vienna, Austria; 4 Hospital Pharmacy, Innsbruck University Hospital, Anichstraße 35, 6020 Innsbruck, Austria

**Keywords:** Competency framework, Hospital pharmacy, Implementation research

## Abstract

**Background:**

Despite the publication of a European wide competency framework for hospital pharmacy by the European Association of Hospital Pharmacist (EAHP) in 2017, not all countries have adopted and implemented such a framework.

**Aim:**

This study aimed to develop and validate a bespoke national hospital pharmacy competency framework for Austria that supports the hospital pharmacy workforce development.

**Method:**

A multi-method study was carried out in three phases. (I) A systematic literature review across 48 websites of healthcare-related associations and six scientific databases was conducted, identifying competency frameworks, guidelines and related documents. (II) Extracted behaviour competencies were reviewed for contextual national appropriateness by three researchers prior to mapping against the “Patient Care and Clinical Pharmacy Skills” domain of European Common Training Framework (CTF). (III) Validation of the resultant draft clinical skills competency framework took place by an expert panel (n = 4; Austrian Association of Hospital Pharmacists (AAHP) board members) discussion. Reporting of findings is aligned with the recommendations for reporting Competency Framework Development in health professions (CONFERD-HP guidelines) and the PRISMA 2020 checklist.

**Results:**

The systematic review (SR) resulted in 28 frameworks, guidelines and related documents and the identification of 379 behaviour competencies, with nineteen mapped to the “Patient Care and Clinical Pharmacy Skills” domain of the CTF (after removal of duplicates). Expert panel discussion resulted in suggested changes to ensure contextual national appropriateness.

**Conclusion:**

This study resulted in the development and validation of the first clinical national pharmacy competency framework for Austria. Future studies should focus on political and practical structures necessary for its successful implementation.

**Supplementary Information:**

The online version contains supplementary material available at 10.1007/s11096-024-01781-3.

## Impact statements

- The methodological approach of adapting the European CTF to national needs may be used by other countries which have a similar development need for clinical pharmacy practice.
- Defining national clinical competencies for hospital pharmacists supports the attainment of the patient safety goals set out by the WHO and the Council of Europe.
- The national framework can be used to inform the progressive development of under- and postgraduate education of clinical pharmacists.
- Planned legislative changes to the scope of hospital pharmacists practice in Austria are dependent on a clear definition of hospital pharmacists’ skills.

## Introduction

A flexible and competent healthcare workforce is imperative for a strong and sustainable healthcare system [1]. According to the World Health Organization (WHO) [2] and the Council of Europe [3] patient safety is now a global priority across all healthcare systems. Therefore, the hospital pharmacy workforce has to be adaptable in order to respond to changes appropriately, which means they are required to have advanced specialist knowledge and skills [4]. Several healthcare-related documents have highlighted the necessity to invest in the global health workforce [5–7]. Several overarching organisations have identified key competencies required to underpin the capability of health care practitioners in general and hospital pharmacists, in particular [8, 9]. According to the National Institute of Health (NIH) “*competencies are the knowledge, skills, abilities, and behaviors that contribute to individual and organizational performance.*” [10]. Given their pivotal role as “experts in medicines”, pharmacists are key in providing medication- and patient safety with competency frameworks forming an inherent part of their training and education for many years [4]. One of the first competency framework for pharmacists was the Global Competency Framework (GbCF) published by the International Pharmaceutical Federation (FIP) in 2012 and updated in 2020 (second version) [8, 11]. Although pharmacy practice may vary between countries, the GbCF is regarded as a set of competencies which are applicable worldwide in order to develop foundation practice within all sectors of pharmacy. As a result, several countries have adapted the GbCF in order to develop their own competency framework based on their own country’s needs [12].

In Europe, the European Association of Hospital Pharmacists (EAHP), published a Common Training Framework (CTF) for hospital pharmacists in 2017 [9]. It was developed to ensure that learning outcomes and achieved competencies are recognised across all European countries [13]. The CTF consists of four domains, namely ‘Patient focus (Patient care and clinical pharmacy skills competencies)’, ‘Medicines focus (Medicines and their use related competencies)’, ‘System focus (Management competencies)’, and ‘Practice focus (Professional competencies)’. It summarises competencies (which consist of a set of behaviour competencies), knowledge, skills, and attitudes, which are required for hospital pharmacists to deliver on and therefore help to fulfill the 44 European Statements of Hospital Pharmacy published by the EAHP in 2014 [13, 14]. Moreover, the CTF clarifies the education that is necessary to underpin the best possible hospital pharmacy practice [13]. Recently, a publication which sought to identify the differences and similarities among the initial pharmacy education and training curricula cross 16 countries worldwide was disseminated [15]. It showed wide variation in education content, focus and correlation between subjects, leading to considerable variations in the skills and competencies of the foundation workforce [15]. Having the CTF provide a further education framework for hospital pharmacists is imperative to “level the playing field” across such a diverse level of foundation education. Some European countries such as the UK have mapped out a further advanced to consultant level framework (ACLF) that sets out the expectations and skills for pharmacists with many years of experience, taking them to consultant level skills [16–18]. Most recently a paper by Meilianti et al. [19] describes the development of a global advanced competency framework as a tool to advance the pharmacy profession globally. Despite these advanced in workforce development, education and continuing professional development (CPD), not all European countries, including Austria, have adopted a competency framework to support and guide their legal and professional development [12].

### Aim

This study aimed to develop and validate a bespoke national hospital pharmacy competency framework that supports the hospital pharmacy workforce development in Austria.

### Ethics approval

Ethical approval was not required as the data were already available in the public domain and did not contain any confidential or commercially sensitive information. According to the ethics committee of the Medical University of Innsbruck an ethical approval for the expert panel discussion was not necessary. Good research practice was followed throughout, and the protocol of the systematic review registered with the International Prospective Register of Systematic Reviews (PROSPERO) [CRD42022323461].

## Method

### Study design

This study used a multi-method approach in three phases: (I) a systematic review of all clinical pharmacy competency frameworks globally; (II) Independent mapping and discursive analysis of contextual national competency appropriateness (CTF); (III) Validation using dicsussion of an expert panel of hospital pharmacists.

#### Systematic review

The systematic review was reported according to ‘Preferred Reporting Items for Systematic Review and Meta-Analysis’ (PRISMA) 2020. The websites of 48 pharmaceutical and/or medical organisations worldwide plus six scientific databases (Guideline Central, PubMed, ScienceDirect, Web of Science, PubPharm and Cochrane Library (Ovid)) were explored for guidelines, frameworks, policies and instructions using a refined search string with the help of a scientific research librarian (Supplementary Tables 1 and 2). Title, abstract and full-text screening of identified documents against the pre-determined inclusion and exclusion criteria was completed by two researchers independently (JTS/AEW). Included were guidelines, frameworks and related documents published by a pharmaceutical and/or medical organization between 2012 and 2022 in the English language containing pharmacy professional related competencies. Discrepancies were resolved by discussion, with a third reviewer (BBM) consulted in case no agreement was reached. The AGREE II Checklist was used to assess the quality and reporting of the competency frameworks [20]. A data extraction form was developed, based on the aims of this study and the four competency domains of the updated GbCF published by FIP in 2020 [11].

#### Mapping and discursive analysis

After extracting all behaviour competencies associated with the clinical/practice skill set (n = 379), these were mapped against the competencies stated in the “Patient Care and Clinical Pharmacy Skills” domain of European Common Training Framework (CTF) by the lead researcher (JTS) and independently checked by BBM and AEW using Word (vs. 2021). A total of 360 behaviour competencies were removed (duplicates (n = 354) between the CTF and one or more other frameworks & n = 6 behaviour competencies for community pharmacists). The remaining behaviour competencies (n = 19) were reviewed for contextual national appropriateness by discursive analysis (JTS/BBM/AEW) and, where appropriate, added to the CTF. If no CTF domain was suitable, the behaviour competency was added to the domain ‘Other’.

#### Validation

Validation of the resultant draft clinical skills competency framework took place by an expert panel discussion consisting of the board members of the Austrian association of hospital pharmacists (AAHP). These experts (n = 4) were chosen as they represented the hospital pharmacists’ workgroup in Austria. They were initially contacted by telephone, during which details of the study were provided. The return of their written feedback was taken as informed consent to partake in the study. The expert panel represented different geographical regions of Austria as well as experience levels (chief pharmacist; pharmacy production lead; practice specialist) and professional body representation (president & vice president of the Austrian Association of Hospital Pharmacists). The experts received the competency framework in advance via email. After they had time to review in detail the framework, an online meeting via Zoom was held and their suggested changes discussed. Consensus was reached for every suggested change to the competency framework. Following validation, the resultant framework was translated into the German language.

### Reflexivity

All authors are pharmacists, two have an extensive background as lead hospital pharmacy practitioners with one having extensive experience in clinical pharmacy research and education as well as a hospital pharmacy practice.

## Results

### Systematic review

#### Identification of records

**Fig. 1:**
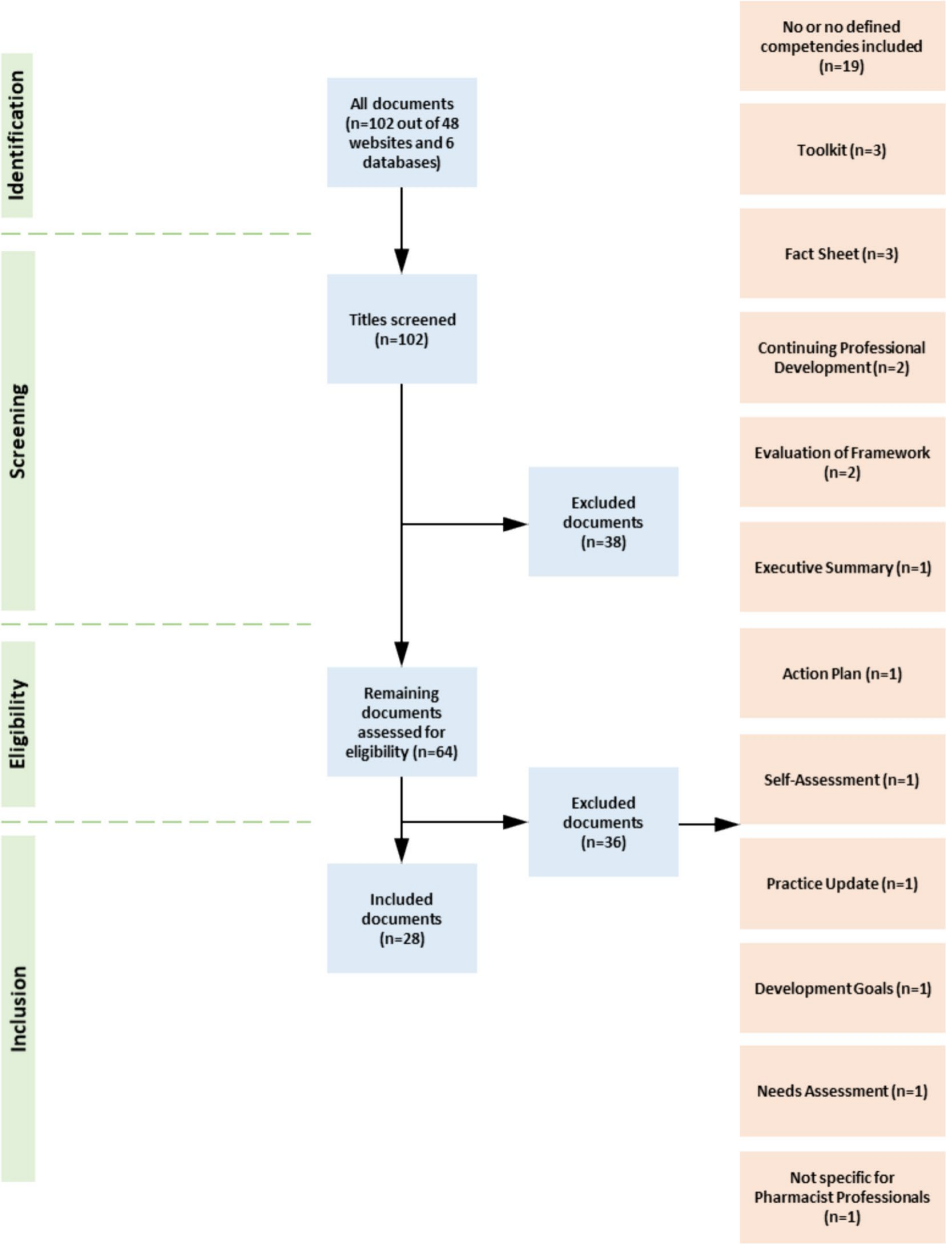
PRISMA flowchart showing the screening process of included professional organisations and databases

Of the 102 documents identified (Professional Organisations n = 96; Databases n = 6), 28 were included (Fig. 1). These documents originated from international pharmaceutical organisations (n = 5) as well as overarching European organisations (n = 5), Australia (n = 12), the UK (n = 1), USA (n = 1) and New Zealand (n = 1). Of these nine were frameworks, one guideline and one tool. In addition, one handbook, one call to action, one reference document and a total of 14 original studies were included (Supplementary Table 3).

#### Quality of included files

Quality assessment was independently undertaken by the principal researcher (JTS) and one other researcher (BBM/AEW). The AGREE II Reporting Checklist could not be applied to 6 out of 28 records and only showed limited applicability to 22 records, therefore the quality of the included documents could not be fully assessed. Practice guidelines rarely reported the ‘Rigour of Development’ (Domain 3), ‘Applicability’ (Domain 5) and ‘Editorial Independence’ (Domain 6). Domain 4 ‘Clarity of Presentation’ was reported infrequently with the most frequently reported domains being ‘Scope and Purpose’ (Domain 1) and ‘Stakeholder Involvement’ (Domain 2).

### Mapping and discursive analysis

#### Selection of competencies and structure of the adapted competency framework

**Fig. 2:**
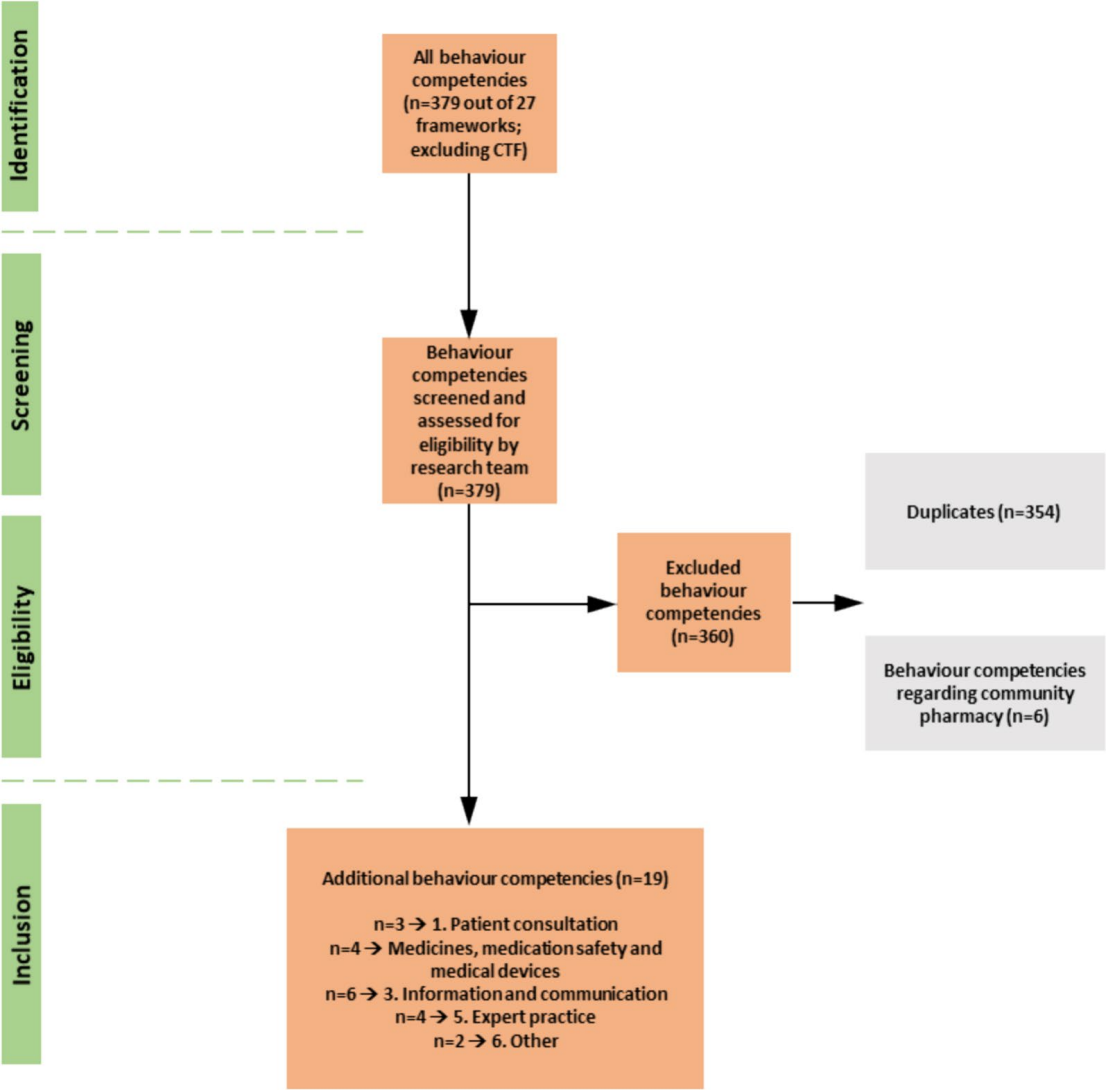
Selection process of behaviour competencies

**Fig. 3:**
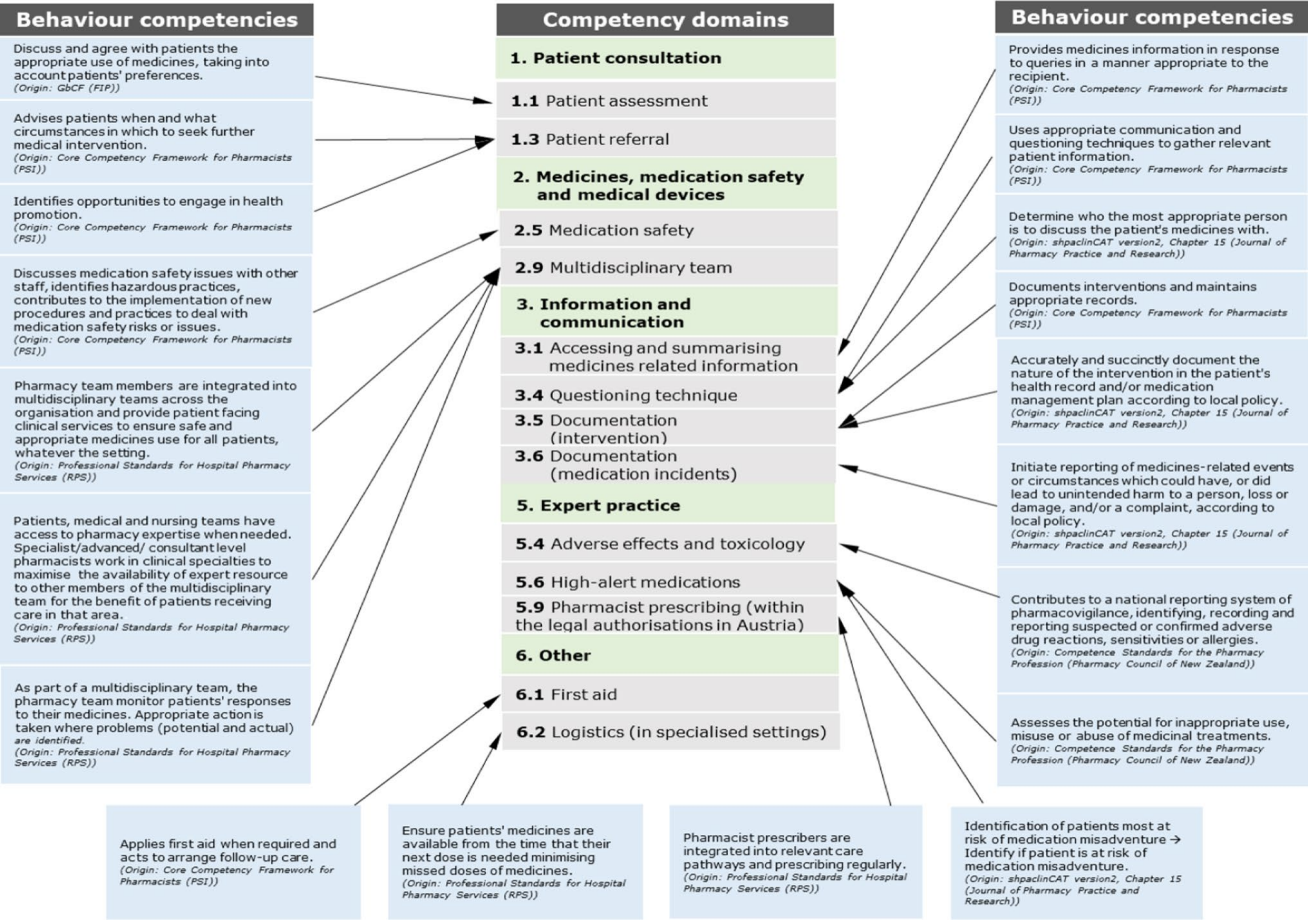
Overview of all 19 added behaviour competencies to the CTF domain 'Patient care and clinical pharmacy skills competencies’

Out of the 27 records (CTF excluded), 379 behaviour competencies for all sectors of pharmacy practice were identified. The research team identified 354 duplicates and six behaviour competencies, which were specific for community pharmacists, not hospital pharmacists, yielding 19 competencies (Fig. 2) which were put forward for discursive analysis to review their contextual national appropriateness and mapping against the “Patient Care and Clinical Pharmacy Skills” domain of European Common Training Framework (CTF) (Fig. 2). Most behaviour competencies (n = 7) originated from the Core Competency Framework for Pharmacists published by the Pharmaceutical Society of Ireland (PSI) [21], followed by five behaviour competencies of the professional standards for hospital pharmacy services by the Royal Pharmaceutical Society of great Britain (RPS) (n = 5) [22], and four behaviour competencies of the clinical competency assessment Tool (shpaclinCAT version 2) by the Society of Hospital Pharmacists of Australia (SHPA) (n = 4) [23]. Three more behaviour competencies were added, of which two originated from the competence standards for the pharmacy profession of New Zealand (n = 2) [24] and one from the GbCF version 3 of FIP (n = 1) [11]. The mapping of all competencies against the CTF domains is illustrated in Fig. 3.

### Validation

#### Suggestions and changes by the expert panel

Changes suggested to the draft clinical competency framework developed in Phase (II) are illustrated in Table 1. It was suggested to change or shorten behaviour competency 1.1.3, as hospital pharmacists in Austria are not allowed to conduct basic physical examination. The research team, together with the expert panel, elected not to change this behaviour competency as this was seen in the context of either conducting basic physical examination in the case of emergency (First Aid) or under delegation of a physician within the legal authorizations in Austria. To avoid confusion between the terms ‘seamless care’ (2.7) and ‘transfer of care’ (2.8), the expert panel suggested adding comments to each term. Therefore, the notes ‘(*focus on medicine management)’ and ‘(*focus on communication)’ were added to 2.7 and 2.8, respectively. Furthermore, another note was added to 6.2 Logistics, namely ‘(in specialised settings), as more detailed logistic aspects are already contained within the “Management” domain of the CTF and was not the focus of the adaptation of the “Patient Care and Clinical Pharmacy Skills” domain. The aspects of prescribing pharmacists and First Aid in the hospital setting was also discussed. As Austrian pharmacists are not yet allowed to prescribe, the comment ‘within the legal authorizations in Austria’ was added this competency (5.9). As every inhabitant of Austria is legally obliged to provide first aid, the expert panel agreed that first aid should be included as a competency for hospital pharmacists (6.1). The final version of the adapted competency framework for Austria is shown in Supplementary file 2.

**Table 1:** Comments made by the expert panel in Phase (III) of the multi-method study on the competencies and illustration of their resolution

Part of the framework	Comments/suggestions by expert panel	Actions and solutions
1. Patient consultation	Should we change or shorten behaviour competency 1.1.3, due to no allowance of hospital pharmacists to do physical examination?	No changes or shortages to 1.1.3 as this was seen in another perspective (within the legal authorisations in Austria).
2. Medicines, medication safety and medical devices:	What is the difference between ‘seamless care’ and ‘transfer of care’?	Comments added to 2.7 Seamless care and 2.8 Transfer of care:
2.7 Seamless care		(*focus on medicine management)
2.8 Transfer of care		(*focus on communication)
5. Expert practice	Pharmacists are not allowed to prescribe in Austria. Why is this aspect included in the framework?	It was aimed to include all additional competencies found worldwide. Therefore, the comment ‘within the legal authorisations in Austria’ was added to competency 5.9. to futureproof against any legal changes.
5.9 Pharmacist prescribing		
6. Other	Is ‘First Aid’ necessary to be included in a competency framework for hospital pharmacists?	The research team agreed on including this aspect as first aid is mandatory by the Austrian law.
6.1 First aid		
6.2 Logistics	Why is there no competency regarding ‘Adequate replacement in the event of supply shortages’ contained in the framework?	More details regarding this aspect are contained in the ‘Management’ part of the CTF. Therefore, a note ‘(in specialised settings)’ was added to 6.2 Logistics.

## Discussion

### Key findings

This study has identified the defined clinical pharmacy competencies needed to help drive and develop hospital pharmacy education and workforce development in Austria. The previous absence of competency framework adoption presented an opportunity to adapt the European hospital pharmacy competency framework (CTF) to align with the national direction for healthcare workforce development for Austria and the aspirations of the WHO and Council of Europe for patient safety.

### Strengths and weaknesses

The adaptation of the European CTF to a bespoke national competency framework for hospital pharmacists in Austria is described in this study. It uses the CONFERD-HP recommendations for reporting competency framework development in health professions [25] and the PRISMA 2020 guidance for reporting systematic reviews. The review protocol was registered with PROSPERO and the competency framework underpinned by European Common Training Framework (CTF) ensuring peer review and robustness in research design. However, the limitations of this study arise from several sources of information not being included. This is as a result of only documents in the English language being included, potentially omitting any bespoke competency frameworks developed in non-English speaking countries. Furthermore, the updated Core Competency Framework for Pharmacists of the PSI and the updated Professional Standards for Hospital Pharmacy Services of the RPS were not included as the review was conducted in early 2022 and the updated competency framework and professional standards version were published in December 2022 and November 2022, respectively. Of note, the first versions of the Core Competency Framework for Pharmacists, as well as the Professional Standards for Hospital Pharmacy Services, are no longer accessible. The lack of standardised wording of competencies made mapping difficult as many were too broad and generic for easy interpretation. The expert panel, while representative of the hospital pharmacy organisation in Austria, was small and may have lacked the benefit of an increased diversity in expertise, potentially biasing the viewpoints and feedback provided on the competency framework. In addition, participants were not entirely representative of all Austrian Federal States. The competency framework provides an overview of necessary (behaviour) competencies for hospital pharmacists and should always be considered within the legal authorisations in Austria.

### Interpretation

The CTF Working Group 3, in which Austria participated, was tasked with "*securing the strong engagement in, and support of, national governments, competent authorities and the European Commission for the project*" [26]. This is an essential step in the implementation of competency frameworks worldwide, as the mere act of developing a competency framework is not enough to evoke a change in overall hospital pharmacy practice. Despite the paucity of robust studies, it is evident that competency frameworks have implications for resources, organisational structures, legal frameworks and education that need to be considered at a country and organisational level [27]. In 2018, the Austrian Ministry of Health, set out its “strategy for patient safety” which clearly mandated the expansion and, where necessary, creation of legal frameworks and requirements to promote a patient safety culture as well as the promotion of education, training and continuing education in the field of patient safety for all healthcare professionals [28]. According to the European Observatory on Health Systems and Policies (2021), Austria has more doctors and nurses than the EU average [29] while having substantially fewer hospital pharmacies and pharmacists than the EU norm [30]. As a result, traditional hierarchical healthcare structures are very ingrained and the scope for a role expansion of hospital pharmacists is curtailed. A recent cross-sectional study investigating the patient safety culture in ten Austrian hospitals, identified significant differences in the perception of patient safety among the 1525 participants from different professional healthcare groups [31]. While it found considerable room for improvement, pharmacy staff were not included in the study, which is symptomatic of the traditional healthcare structures, where pharmacists are strongly associated with more traditional roles such as logistics, procurement and production and less so with medication and patient safety [32]. This is mirrored in many other central European countries who are equally struggling to evolve into more patient facing roles [33, 34]. As a result, countries need to invest in stakeholder analysis studies to identify how competency frameworks can be implemented into existing national healthcare structures and education.

Reforms to the global pharmacy education standards and the development of supporting competency frameworks for lifelong learning are now being used to regulate career entry, benchmark standards of practice and facilitate expertise development [27, 35, 36]. Competency frameworks help to reshape (further-) education by defining the competencies and skills needed by hospital pharmacists and adapting the education and training accordingly [37, 38]. Austria has a further education program allowing licensed pharmacists to become specialist hospital pharmacist (aHPh). The aim of this specialist three-year part-time education program is to impart advanced knowledge and practical skills in hospital pharmacy (specialisation) [39]. The curriculum content is decided upon by the further education commission and includes clinical pharmacy, production and management topics, however it is not stated if it aligns with any of the European or global pharmacy competency frameworks [39]. Given the emphasis of the pharmacy profession worldwide to develop a competent and adaptable pharmacy workforce, more has to be done to offer competency based driven education and assessments [40, 41]. A current lack of robust implementation and success data for competency-based pharmacy education calls for more studies identifying the needs of patients, learners, healthcare systems and that of academic institutions, especially in the face of fast paced technological changes [42].

This clinical pharmaceutical competency framework provides the necessary basis for the expansion of the legal and professional practice of hospital pharmacists and the amendment to the Austrian Pharmacy Act by the Health Ministry. The competency framework is currently being considered by both the AAHP and the Austrian Chamber of Pharmacists, for its potential contribution to further legal advancement of the Austrian hospital pharmacists role description.

### Further research

Obtaining views from all other stakeholder groups (patients, medical and pharmaceutical organisations, government and non-government organisations, healthcare insurance agencies etc.) will be required to determine implementation barriers and facilitators within the Austrian national healthcare service. Obtaining the view of the hospital pharmacist workforce may also provide further validity to the bespoke competency framework developed.

## Conclusion

A comprehensive development process was undertaken to create a bespoke national clinical pharmacy competency framework for hospital pharmacists in Austria. This competency framework, which is underpinned by the European Common Training Framework (CTF), will hopefully provide the necessary foundation to expand hospital pharmacists’ legal and professional practice as well as help to reshape hospital pharmacy education in a bid to align with the ambitions for healthcare workforce development and patient safety. Views from stakeholder groups are imperative to facilitate successful implementation.

## Supplementary Information

Below is the link to the electronic supplementary material.

Supplementary file1 (DOCX 49 KB)

Supplementary file2 (DOCX 26 KB)

## References

[1] Anand S, Bärnighausen T. Human resources and health outcomes: cross-country econometric study. Lancet. 2004;364(9445):1603–9. 10.1016/S0140-6736(04)17313-3. 15519630 10.1016/S0140-6736(04)17313-3

[2] World Health Organization (WHO). Global patient safety action plan 2021–2030: towards eliminating avoidable harm in health care. 2021. Available from: https://apps.who.int/iris/rest/bitstreams/1360307/retrieve. Accessed 13 Feb 2024.

[3] Council of Europe. Resolution CM/Res (2020) 3 on the implementation of pharmaceutical care for the benefit of patients and health services. (Adopted by the committee of ministers on 11 March 2020 at the 1370th meeting of the Ministers’ Deputies). 2020. Available from: https://rm.coe.int/09000016809cdf26. Accessed 26 Feb 2024.

[4] International Pharmaceutical Federation (FIP). FIP Global Advanced Development Framework: supporting the advancement of the profession version 1. The Hague: International pharmaceutical federation. 2020. Available from: https://www.fip.org/search?page=gadf. Accessed 13 Feb 2024.

[5] World Health Organization (WHO). Global strategy on human resources for health: Workforce 2030. Geneva: WHO. 2016. Available from: https://www.who.int/publications/i/item/9789241511131. Accessed 13 Feb 2024.

[6] World Health Organization (WHO). Final report of the expert group to the high-level commission on health employment and economic growth. Geneva: WHO. 2016. Available from: https://www.who.int/publications/i/item/9789241511285. Accessed 13 Feb 2024.

[7] World Health Organization (WHO). “Working for Health”: A Five-year action plan for health employment and inclusive economic growth (2017–21). Geneva: WHO. 2017. Available from: https://www.who.int/publications/i/item/9789241514149. Accessed 13 Feb 2024.

[8] International Pharmaceutical Federation (FIP). FIP education initiatives pharmacy education taskforce a global competency framework version 1. 2012. Available from: https://www.fip.org/publications?publicationCategory=6&publicationYear=2012&publicationKeyword=. Accessed 13 Feb 2024.

[9] European Association of Hospital Pharmacists (EAHP). Common training framework (CTF). 2017. Available from: https://www.hospitalpharmacy.eu/competency-framework. Accessed 13 Feb 2024.

[10] National Health Institute (NIH). What are competencies? No date. Available from: https://hr.nih.gov/about/faq/working-nih/competencies/what-are-competencies.

[11] International Pharmaceutical Federation (FIP). Executive summary FIP global competency framework supporting the development of foundation and early career pharmacists version 2. 2020. Available from: https://www.fip.org/publications?publicationCategory=6&publicationYear=&publicationKeyword=. Accessed: 13 Feb 2024.

[12] International Pharmaceutical Federation (FIP). FIP*Ed* Global Education Report. 2013. Available from: https://www.fip.org/publications?publicationCategory=6&publicationYear=&publicationKeyword=. Accessed 13 Feb 2024.

[13] European Association of Hospital Pharmacists (EAHP). What is CTF? No date. Available from: https://www.hospitalpharmacy.eu/general-information. Accessed 13 Feb 2024.

[14] The European Statements of Hospital Pharmacy. Eur J Hosp Pharm. 2014;21:256–8. 10.1136/ejhpharm-2014-000526.

[15] Arakawa N, Bruno-Tomé A, Bates I. A global comparison of initial pharmacy education curricula: an exploratory study. Innov Pharm. 2022;11(1):18. 10.24926/iip.v11i1.2093. 10.24926/iip.v11i1.2093 PMC8132530 34017634

[16] Competency Development Evaluation Group (CoDEG). Advanced to consultant level framework (ACLF). 2009. Available from: https://www.codeg.org/advanced-level-practice/developing-the-aclf/index.html. Accessed 13 Feb 2024.

[17] Royal Pharmaceutical Society (RPS). Core advanced pharmacist curriculum. No date. Available from: https://www.rpharms.com/Portals/0/Credentialing/RPS%20-%20Core%20Advanced%20curriculumFINAL.pdf?ver=iR3AZBxZA79vddgs6a6wUQ%3d%3d. Accessed 13 Feb 2024.

[18] Royal Pharmaceutical Society (RPS). Consultant pharmacist curriculum. No date. Available from: https://www.rpharms.com/Portals/0/Consultant/Open%20Access/RPS%20Consultant%20Pharmacist%20Curriculum%202020_FINAL.pdf?ver=-TmAIYQLYxE5Xh924jA0MA%3d%3d. Accessed: 13 Feb 2024.

[19] Meilianti S, Galbraith K, Bader L, et al. The development and validation of a global advanced development framework for the pharmacy workforce: a four-stage multi-methods approach. Int J Clin Pharm. 2023;45:940–51. 10.1007/s11096-023-01585-x. 37179511 10.1007/s11096-023-01585-x PMC10366019

[20] AGREE Next Steps Consortium (2017). The AGREE II Instrument [Electronic version]. Available from: https://www.agreetrust.org/wp-content/uploads/2017/12/AGREE-II-Users-Manual-and-23-item-Instrument-2009-Update-2017.pdf. Accessed 13 Feb 2024.

[21] The Pharmaceutical Society of Ireland (PSI). Core competency framework for pharmacists, version2. 2022. Available from: https://www.thepsi.ie/gns/Pharmacy_Practice/core-competency-framework.aspx. Accessed 05 Jun 2024.

[22] Royal Pharmaceutical Society (RPS). Professional standards for hospital pharmacy services, version 4. 2022. Available from: https://www.rpharms.com/Portals/0/RPS%20document%20library/Open%20access/Hospital%20Standards/PRS-Professional%20Standards%20for%20Hospital%20Pharmacy%20Services_amend-221212.pdf. Accessed 05 Jun 2024.

[23] Chapter 15. Clinical competency assessment tool (shpaclinCAT version 2). J Pharm Pract Res. 2013 43:50–67. 10.1002/j.2055-2335.2013.tb00909.x

[24] Pharmacy Council of New Zealand. Safe effective pharmacy practice competence standards for the pharmacy profession. 2015. Available from: https://pharmacycouncil.org.nz/wp-content/uploads/2021/04/CompStds2015Web.pdf. Accessed 13 Feb 2024.

[25] Batt AM, Tavares W, Horsley T, et al. CONFERD-HP collaborators CONFERD-HP: recommendations for reporting competency framework development in health professions. Br J Surg. 2023;110(2):233–41. 10.1093/bjs/znac394. 36413510 10.1093/bjs/znac394 PMC10364529

[26] European Association of Hospital Pharmacists (EAHP). Who is involved. No date. Available from: https://www.hospitalpharmacy.eu/who-is-involved. Accessed 13 Feb 2024.

[27] Udoh A, Bruno-Tomé A, Ernawati D, et al. The development, validity and applicability to practice of pharmacy-related competency frameworks: a systematic review. Res Social Adm Pharm. 2021;17(10):1697–718. 10.1016/j.sapharm.2021.02.014. 33640334 10.1016/j.sapharm.2021.02.014

[28] Bundesministerium für Arbeit, Soziales, Gesundheit und Konsumentenschutz. Patientensicherheitsstrategie 2.0 Eine österreichweite Rahmenvorgabe (Patient Safety Strategy 2.0 An Austria-wide framework). 2018. Available from: https://www.sozialministerium.at/Themen/Gesundheit/Gesundheitssystem/Gesundheitssystem-und-Qualitaetssicherung/Patient-innensicherheit-und-Patient-inneninformationen/Patientensicherheitsstrategie-2.0.html. Accesseed 13 Feb 2024.

[29] OECD/European Observatory: Health Systems and Policies Österreich: Länderprofil Gesundheit 2019. 2019.State of Health in the EU, OECD Publishing, Paris/European Observatory on Health Systems and Policies, Brussels. Available from: https://health.ec.europa.eu/system/files/2021-12/2021_chp_at_german.pdf. Accessed 13 Feb 2024.

[30] Langer T, Spreitzer H, Ditfurth T, et al. Pharmacy practice and education in Austria. Pharm (Basel). 2018;6(3):55. 10.3390/pharmacy6030055. 10.3390/pharmacy6030055 PMC6164448 29949941

[31] Draganović Š, Offermanns G. Patient safety culture in Austria and recommendations of evidence-based instruments for improving patient safety. PLoS ONE. 2022;17(10): e0274805. 10.1371/journal.pone.0274805. 36251643 10.1371/journal.pone.0274805 PMC9576070

[32] Stemer G, Laml-Wallner G, Kuegler I, et al. Comprehensive evaluation of clinical pharmacists’ interventions in a large Austrian tertiary care hospital. Eur J Hosp Pharm. 2012;19:529–34. 10.1136/ejhpharm-2012-000131.

[33] Atkinson J. Advances in pharmacy practice: a look towards the future. Pharm (Basel). 2022;10(5):125. 10.3390/pharmacy10050125. 10.3390/pharmacy10050125 PMC9608826 36287446

[34] Pearson GJ. Evolution in the practice of pharmacy–not a revolution! Can Med Assoc J. 2007;176(9):1295–6. 10.1503/cmaj.070041. 17452664 10.1503/cmaj.070041 PMC1852869

[35] International Pharmaceutical Federation (FIP). FIP Global Competency Framework (GbCFv2) handbook Supporting early career training strategy. 2023. Available from: https://www.fip.org/file/5546. Accessed 13 Feb 2024.

[36] European Association of employed community pharmacists in Europe (EPhEU). Pharmacy in Europe. No date. Available from: https://epheu.eu/pharmacy-in-europe/. Accessed 13 Feb 2024.

[37] International Pharmaceutical Federation (FIP). Competency-based education in pharmacy and pharmaceutical sciences A FIP handbook to support implementation of competency-based education and training. 2022. Available from: https://www.fip.org/file/5338. Accessed 13 Feb 2024.

[38] Chamoun N, Ramia E, Sacre H, et al. Validation of the specialized competency framework for pharmacists in hospital settings (SCF–PHS): a cross-sectional study. J Pharm Policy Pract. 2023;16:86. 10.1186/s40545-023-00592-7. 37430355 10.1186/s40545-023-00592-7 PMC10332012

[39] Österreichische Apothekerkammer. Verordnung der Österreichischen Apothekerkammer betreffend die Weiterbildung zur Fachapothekerin oder zum Fachapotheker für Krankenhauspharmazie (Regulation of the Austrian Chamber of Pharmacists concerning further training to become a specialised pharmacist for hospital pharmacy). 2015. Available from: https://www.apothekerkammer.at/infothek/rechtliche-hintergruende/apothekerkammer-und-apothekerberufsrecht/weiterbildungsordnung-krankenhausfachapotheker-khfa-wbo-2015. Accessed 13 Feb 2024.

[40] Austin Z, Andriole DA, Rhoney DH. Is it Time for competency-based education to move forward in pharmacy education? Am J Pharm Educ. 2023;87(10):100550. 10.1016/j.ajpe.2023.100550. 37331516 10.1016/j.ajpe.2023.100550

[41] European Association of Faculties of Pharmacy (EAFP). EAFP Position Paper 2018. 2018. Available from: https://eafponline.eu/wp-content/uploads/2018/06/here.pdf. Accessed 13 Feb 2024.

[42] Weidmann AE. Artificial intelligence in academic writing and clinical pharmacy education: consequences and opportunities. Int J Clin Pharm. 2024;46(3):751–4. 10.1007/s11096-024-01705-1. 38472596 10.1007/s11096-024-01705-1 PMC11133206

