# Supplementary material for: Development and validation of a national clinical pharmacy competency framework for hospital pharmacists in Austria: a multi-method study
Source: Int J Clin Pharm. 2024 Aug 7;46(6):1400–9. doi: 10.1007/s11096-024-01781-3 (PMC11576871; doi:10.1007/s11096-024-01781-3)
Supplement: Supplementary file 1 — Supplementary file1 (DOCX 49 KB) [file 11096_2024_1781_MOESM1_ESM.docx]

Supplementary material for:

**Development and validation of a national clinical pharmacy competency framework for hospital pharmacists: a multi-method study**

Stoll, J. T.^1^; Böhmdorfer-McNair, B.^2,3^; Jeske, M.^4^; Weidmann, A. E.^1^

^1^ *Department of Clinical Pharmacy, Innsbruck University, Innrain 15, 6020 Innsbruck, Austria; corresponding author: jasmin.stoll@uibk.ac.at*

^2^ *Hospital Pharmacy, Hietzing Clinic, Vienna Health Association, Wolkersbergenstraße 1, 1130 Vienna, Austria,*

*^3^ Karl Landsteiner Institute for Clinical Risk Management, 1130 Vienna, Austria*

*^4^ Hospital Pharmacy, Innsbruck University Hospital, Anichstraße 35, 6020 Innsbruck, Austria*

Supplementary Table 1.: Included organizations, number of hits and key words used for the search in Phase I (SR) of this mixed methods research

| Professional Organisation | Country/Origin | Website | Number of hits | Key words used | |
| --- | --- | --- | --- | --- | --- |
| FIP (International Pharmaceutical Federation) | International | <https://www.fip.org/> | 18 | Competency, skill, ability, proficiency, capability, expertise,  pharmacist, clinical pharmacist, hospital pharmacist, ‘advanced pharmacist, consultant pharmacist, hospital, clinic, medical center, sanatorium, infirmary, medical institution,  drug therapy safety, medication therapy safety, medicine therapy safety, patient safety, harm, drug safety, medication safety, medicine safety, ‘prescribing error’), guidelines, framework, instruction, protocol, guidance, standard procedure, standard | |
| ISHP (International Society for the History of Pharmacy) | International | <https://histpharm.org/> | 0 |  |  |
| WHO (World Health Organisation) | International | <https://www.who.int/> | 3 |  |  |
| PGEU (Pharmaceutical Group of the European Union) | Europe | <https://www.pgeu.eu/> | 0 |  |  |
| EAHP (European Association of Hospital Pharmacists) | Europe | <https://www.eahp.eu/> | 1 |  |  |
| ESCP (European Society of Clinical Pharmacy) | Europe | [Home Page (escpweb.org)](https://escpweb.org/) | - No or no suitable search function |  |  |
| AAHP (Austrian Association of Hospital Pharmacists) | Austria | <https://www.aahp.at/> | 0 |  |  |
| Österreichische Apothekerkammer | Austria | <https://www.apothekerkammer.at/> | 0 |  |  |
| APB (Association Pharmaceutique Belge) | Belgium | <https://www.apb.be/fr/corp/Pages/default.aspx> | - No or no suitable search function |  |  |
| Pharmadanmark (The Danish Association of Pharmacists) | Denmark | <https://pharmadanmark.dk/en/vacancies-pharmacy-sector> | 0 |  |  |
| Ordre National des Pharmaciens | France | <http://www.ordre.pharmacien.fr/> | - Not in the English language |  |  |
| ABDA (Bundesvereinigung Deutscher Apothekerverbände) | Germany | <https://www.abda.de/> | 1 |  |  |
| ADKA (Association of German Hospital Pharmacists) | Germany | <https://www.adka.de/en/> | 1 |  |  |
| PSI (Pharmaceutical Society of Ireland) | Ireland | <https://www.thepsi.ie/gns/home.aspx> | 5 |  |  |
| Norwegian Pharmacy Association | Norway | <http://www.apotek.no/> | - Not in the English language |  |  |
| ANF (Associação Nacional de Farmácias) | Portugal | [www.grupoanf.pt](https://www.grupoanf.pt/?msclkid=a733afbdd04111ec9883ec4f6df4c435) | Website not available |  |  |
| Infarmed (Instituto Nacional da Farmácia e do Medicamento) | Portugal | <https://www.infarmed.pt/> | 0 |  |  |
| Ordem dos Farmacêuticos | Portugal | <https://www.ordemfarmaceuticos.pt/pt/> | 0 |  |  |
| PSNI (Pharmaceutical Society of Northern Ireland) | UK | <https://www.psni.org.uk/> | 5 |  |  |
| RPS (Royal Pharmaceutical Society) | UK | <https://www.rpharms.com/> | 4 |  |  |
| The Academy of Pharmaceutical Sciences | UK | <https://www.apsgb.co.uk/> | - No or no suitable search function |  |  |
| BSHP (British Society for the History of Pharmacy) | UK | <https://www.bshp.org/> | - No or no suitable search function |  |  |
| CPPE (Centre for Pharmacy Postgraduate Education) | UK | <https://www.cppe.ac.uk/> | 4 |  |  |
| GHP (Guild of Healthcare Pharmacists) | UK | <https://www.ghp.org.uk/> | 0 |  |  |
| UKCPA (United Kingdom Clinical Pharmacy Association) | UK | <https://ukclinicalpharmacy.org/> | 2 |  |  |
| NICE (National Institute for Health and Care Excellence) | UK | <https://www.nice.org.uk/> | 0 |  |  |
| APhA (American Pharmacists Association) | USA | <https://www.pharmacist.com/> | 0 |  |  |
| ASCP (American Society of Consultant Pharmacists) | USA | <https://www.ascp.com/default.aspx> | 0 |  |  |
| ASHP (American Society of Health-System Pharmacists) | USA | <https://www.ashp.org/?login> | 0 |  |  |
| ACCP (American College of Clinical Pharmacy) | USA | <https://www.accp.com/> | 2 |  |  |
| CPNP (College of Psychiatric and Neurologic Pharmacists) | USA | <https://cpnp.org/> | 0 |  |  |
| AMCP (Academy of Managed Care Pharmacy) | USA | <https://www.amcp.org/> | 0 |  |  |
| ASMSO (American Society of Medication Safety Officers) | USA | <https://www.medsafetyofficer.org/> | - No or no suitable search function |  |  |
| NPhA (National Pharmaceutical Association) | USA | <https://nationalpharmaceuticalassociation.org/> | - No or no suitable search function |  |  |
| NCAP (North Carolina Association of Pharmacists) | USA | <https://www.ncpharmacists.org/> | 1 |  |  |
| NAPRA (National Association of Pharmacy Regulatory Authorities) | Canada | <https://www.napra.ca/> | 8 |  |  |
| CPhA (Canadian Pharmacists Association) | Canada | <https://www.pharmacists.ca/> | 1 |  |  |
| CSHP (Canadian Society of Hospital Pharmacists) | Canada | <https://www.cshp.ca/> | 0 |  |  |
| BCPhA (British Columbia Pharmacy Association) | Canada | <https://www.bcpharmacy.ca/> | 0 |  |  |
| BCCP (British Columbia College of Pharmacists) | Canada | <https://www.bcpharmacists.org/> | 0 |  |  |
| OCP (Ontario College of Pharmacists) | Canada | <https://www.ocpinfo.com/> | 7 |  |  |
| OPQ (Ordre des Pharmaciens du Québec) | Canada | <https://www.opq.org/> | - Not in the English language |  |  |
| Pharmaceutical Association of Israel | Israel | <https://www.pharmacy.org.il/> | - No or no suitable search function |  |  |
| ACP (Australasian College of Pharmacy) | Australia/Asia | <https://www.acp.edu.au/> | 1 |  |  |
| PSA (Pharmaceutical Society of Australia) | Australia | <https://www.psa.org.au/> | 6 |  |  |
| PGA (Pharmacy Guild of Australia) | Australia | <https://www.guild.org.au/> | 0 |  |  |
| SHPA (Society of Hospital Pharmacists of Australia) | Australia | <https://www.shpa.org.au/> | 23 |  |  |
| PSNZ (Pharmaceutical Society of New Zealand Incorporated) | New Zealand | <https://www.psnz.org.nz/> | 3 |  |  |
| Total number of professional organisations (n=48) Total number of documents (n=96) | | | |  |  |

Supplementary Table 2.: Included databases, number of hits, total number of hits and search string used in Phase I (SR) of this mixed methods research

| Databases | Website | Number of hits | Search string used |
| --- | --- | --- | --- |
| Guideline Central | <https://www.guidelinecentral.com/> | 0 | (competenc* OR skills OR ability OR proficiency OR capability OR expertise) AND  (pharmacist OR ‘clinical pharmacist’ OR ‘hospital pharmacist’ OR ‘advanced pharmacist’ OR ‘consultant pharmacist’) AND  (hospital OR clinic OR medical cent* OR sanatorium OR infirmary OR ‘medical institution’) AND  (‘drug therapy safety’ OR ‘medication therapy safety’ OR ‘medicine therapy safety’ OR ‘patient safety’ OR harm OR ‘drug safety’ OR ‘medication safety’ OR ‘medicine safety’ OR ‘prescribing error’) AND  (guidelines OR framework OR instruction OR protocol OR guidance OR standard procedure OR standard) AND  (Clinical Competence / standards OR Pharmacy Service, Hospital / standards) |
| PubMed | <https://pubmed.ncbi.nlm.nih.gov/> | 2 |  |
| Science Direct | <https://www.sciencedirect.com/> | 4 |  |
| Web of Science | <https://www.webofscience.com/wos/woscc/basic-search> | 0 |  |
| PubPharm | <https://www.pubpharm.de/de> | 0 |  |
| Cochrane Library (Ovid) | <https://tools.ovid.com/ovidtools/cochrane.html> | 0 |  |
| Total number of databases (n=6) Total number of database hits (n=6) | | |  |

Supplementary Table 3.: Summary of the 28 included competency documents and selected competencies added to the bespoke Austrian framework for hospital pharmacy practice

| Included documents (n=28) | |  |  |  | | |  |  |  |
| --- | --- | --- | --- | --- | --- | --- | --- | --- | --- |
| Title | **Organization/Authors/**  **Journal** | | **Year of publication** | **Origin/Country of origin** | Type of file (framework, guideline, etc.) | | Quality assessment (AGREE II) | Behaviour competency (included in the bespoke framework) | |
| FIP Education Initiatives Pharmacy Education Taskforce: A Global Competency Framework Version 1 - GbCF | FIP | | 2012 | International | Competency Framework | Limited applicability to AGREE II Reporting Checklist | | No competencies added | |
| Executive Summary  FIP Global Competency  Framework  Supporting the development of  foundation and early career  pharmacists  Version 2 - GbCF | FIP | | 2020 | International | Competency Framework | Limited applicability to AGREE II Reporting Checklist | | Discuss and agree with patients the appropriate use of medicines, taking into account patients' preferences. | |
| FIP Global Advanced Development Framework Handbook  Supporting advancement of the profession  Version 1 - GADF | FIP | | 2020 | International | Framework, handbook | Limited applicability to AGREE II Reporting Checklist | | No competencies added | |
| FIP Global Call to Action for Advancing Pharmaceutical Education | FIP | | 2021 | International | Call to action | Limited applicability to AGREE II Reporting Checklist | | No competencies added | |
| Patient safety  Pharmacists’ role in  “Medication without  harm” | FIP | | 2020 | International | Reference document | Limited applicability to AGREE II Reporting Checklist | | No competencies added | |
| Common Training Framework (CTF) | European Association of Hospital Pharmacists (EAHP) | | 2017 | Europe | Competency framework | | No applicability to AGREE II Reporting Checklist | Not applicable; used as a basis for national framework development | |
| **Core Competency Framework for Pharmacists** | Pharmaceutical Society of Ireland (PSI) | | 2013 | Ireland | Competency framework | Limited applicability to AGREE II Reporting Checklist | | Advises patients when and what circumstances in which to seek further medical intervention. | |
|  |  | |  |  |  | |  | Identifies opportunities to engage in health promotion. | |
|  |  | |  |  |  |  | | Discusses medication safety issues with other staff, identifies hazardous practices, contributes to the implementation of new procedures and practices to deal with medication safety risks or issues. | |
|  |  | |  |  |  |  | | Provides medicines information in response to queries in a manner appropriate to the recipient. | |
|  |  | |  |  |  |  | | Uses appropriate communication and questioning techniques to gather relevant patient information. | |
|  |  | |  |  |  |  | | Documents interventions and maintains appropriate records. | |
|  |  | |  |  |  |  | | Applies first aid when required and acts to arrange follow-up care. | |
| Professional Standards  for Hospital Pharmacy Services | Royal Pharmaceutical Society (RPS) | | 2017 | UK | Competency framework | Limited applicability to AGREE II Reporting Checklist | | Pharmacy team members are integrated into multidisciplinary teams across the organisation and provide patient facing clinical services to ensure safe and appropriate medicines use for all patients, whatever the setting. | |
|  |  | |  |  |  |  | | Patients, medical and nursing teams have access to pharmacy expertise when needed. Specialist/advanced/ consultant level pharmacists work in clinical specialties to maximise the availability of expert resource to other members of the multidisciplinary team for the benefit of patients receiving care in that area. | |
|  |  | |  |  |  |  | | As part of a multidisciplinary team, the pharmacy team monitor patients' responses to their medicines. Appropriate action is taken where problems (potential and actual) are identified. | |
|  |  | |  |  |  |  | | Pharmacist prescribers are integrated into relevant care pathways and prescribing regularly. | |
|  |  | |  |  |  |  | | Ensure patients' medicines are available from the time that their next dose is needed minimising missed doses of medicines. | |
| **The RPS Advanced Pharmacy Framework (APF)** | Royal Pharmaceutical Society (RPS) | | 2013 | UK | Competency framework | Limited applicability to AGREE II Reporting Checklist | | No competencies added | |
| Competency development for pharmacy: Adopting and adapting the Global Competency Framework | Asmaa Al-Haqan et al.; Research in Social and Administrative Pharmacy | | 2021 | UK | Original study related to competency framework development | No applicability to AGREE II Reporting Checklist | | No competencies added | |
| The RPS  Foundation Pharmacist  Framework | Royal Pharmaceutical Society (RPS) | | 2019 | UK | Competency framework | Limited applicability to AGREE II Reporting Checklist | | No competencies added | |
| ACCP Clinical Pharmacist Competencies | American College of Clinical Pharmacy (ACCP) | | 2017 | USA | Guideline | Limited applicability to AGREE II Reporting Checklist | | No competencies added | |
| Chapter 1: Medication Reconciliation | Society of Hospital Pharmacists of Australia (SHPA)  Journal of Pharmacy Practice and Research Volume 43, No. 2 (suppl) | | 2013 | Australia | Original study related to competency frameworks | Limited applicability to AGREE II Reporting Checklist | | No competencies added | |
| Chapter 2: Assessment of Current Medication Management | Society of Hospital Pharmacists of Australia (SHPA)  Journal of Pharmacy Practice and Research Volume 43, No. 2 (suppl) | | 2013 | Australia | Original study related to competency frameworks | Limited applicability to AGREE II Reporting Checklist | | No competencies added | |
| Chapter 3: Clinical Review, Therapeutic Drug Monitoring and  Adverse Drug Reaction Management | Society of Hospital Pharmacists of Australia (SHPA)  Journal of Pharmacy Practice and Research Volume 43, No. 2 (suppl) | | 2013 | Australia | Original study related to competency frameworks | Limited applicability to AGREE II Reporting Checklist | | No competencies added | |
| Chapter 4: Medication Management Plan | Society of Hospital Pharmacists of Australia (SHPA)  Journal of Pharmacy Practice and Research Volume 43, No. 2 (suppl) | | 2013 | Australia | Original study related to competency frameworks | Limited applicability to AGREE II Reporting Checklist | | No competencies added | |
| Chapter 5: Providing Medicines Information | Society of Hospital Pharmacists of Australia (SHPA)  Journal of Pharmacy Practice and Research Volume 43, No. 2 (suppl) | | 2013 | Australia | Original study related to competency frameworks | Limited applicability to AGREE II Reporting Checklist | | No competencies added | |
| Chapter 6: Facilitating Continuity of Medication Management on Transition Between Care Settings | Society of Hospital Pharmacists of Australia (SHPA)  Journal of Pharmacy Practice and Research Volume 43, No. 2 (suppl) | | 2013 | Australia | Original study related to competency frameworks | Limited applicability to AGREE II Reporting Checklist | | No competencies added | |
| Chapter 7: Participating in Interdisciplinary Care Planning | Society of Hospital Pharmacists of Australia (SHPA)  Journal of Pharmacy Practice and Research Volume 43, No. 2 (suppl) | | 2013 | Australia | Original study related to competency frameworks | Limited applicability to AGREE II Reporting Checklist | | No competencies added | |
| Chapter 8: Prioritising Clinical Pharmacy Services | Society of Hospital Pharmacists of Australia (SHPA)  Journal of Pharmacy Practice and Research Volume 43, No. 2 (suppl) | | 2013 | Australia | Original study related to competency frameworks | Limited applicability to AGREE II Reporting Checklist | | No competencies added | |
| Chapter 9: Staffing Levels and Structure for the Provision of Clinical Pharmacy Services | Society of Hospital Pharmacists of Australia (SHPA)  Journal of Pharmacy Practice and Research Volume 43, No. 2 (suppl) | | 2013 | Australia | Original study related to competency frameworks | Limited applicability to AGREE II Reporting Checklist | | No competencies added | |
| Chapter 10: Training and Education | Society of Hospital Pharmacists of Australia (SHPA)  Journal of Pharmacy Practice and Research Volume 43, No. 2 (suppl) | | 2013 | Australia | Original study related to competency framework | Limited applicability to AGREE II Reporting Checklist | | No competencies added | |
| Chapter 11: Participating in Research | Society of Hospital Pharmacists of Australia (SHPA)  Journal of Pharmacy Practice and Research Volume 43, No. 2 (suppl) | | 2013 | Australia | Original study related to competency frameworks | No applicability to AGREE II Reporting Checklist | | No competencies added | |
| Chapter 13: Documenting Clinical Activities | Society of Hospital Pharmacists of Australia (SHPA)  Journal of Pharmacy Practice and Research Volume 43, No. 2 (suppl) | | 2013 | Australia | Original study related to competency frameworks | No applicability to AGREE II Reporting Checklist | | No competencies added | |
| Chapter 14: Improving the Quality of Clinical Pharmacy Services | Society of Hospital Pharmacists of Australia (SHPA)  Journal of Pharmacy Practice and Research Volume 43, No. 2 (suppl) | | 2013 | Australia | Original study related to competency frameworks | No applicability to AGREE II Reporting Checklist | | No competencies added | |
| Chapter 15: Clinical Competency Assessment Tool (shpaclinCAT version 2) | Society of Hospital Pharmacists of Australia (SHPA)  Journal of Pharmacy Practice and Research Volume 43, No. 2 (suppl) | | 2013 | Australia | Clinical Competency Assessment Tool | No applicability to AGREE II Reporting Checklist | | Determine who the most appropriate person is to discuss the patient's medicines with. | |
|  |  | |  |  |  |  | | Accurately and succinctly document the nature of the intervention in the patient's health record and/or medication management plan according to local policy. | |
|  |  | |  |  |  |  | | Initiate reporting of medicines-related events or circumstances which could have, or did lead to unintended harm to a person, loss or damage, and/or a complaint, according to local policy. | |
|  |  | |  |  |  |  | | Identification of patients most at risk of medication misadventure 🡪 Identify if patient is at risk of medication misadventure. | |
| National Competency  Standards Framework  for Pharmacists  in Australia (NCSF) | Pharmaceutical Society of Australia (PSA) | | 2016 | Australia | Competency framework | Limited applicability to AGREE II Reporting Checklist | | No competencies added | |
| Safe Effective Pharmacy Practice  Competence Standards for the Pharmacy Profession | Pharmacy Council of New Zealand | | 2015 | New Zealand | Competency framework | Limited applicability to AGREE II Reporting Checklist | | Contributes to a national reporting system of pharmacovigilance, identifying, recording and reporting suspected or confirmed adverse drug reactions, sensitivities or allergies. | |
|  |  | |  |  |  |  | | Assesses the potential for inappropriate use, misuse or abuse of medicinal treatments. | |
