# Supplementary material for: Development and validation of a national clinical pharmacy competency framework for hospital pharmacists in Austria: a multi-method study
Source: Int J Clin Pharm. 2024 Aug 7;46(6):1400–9. doi: 10.1007/s11096-024-01781-3 (PMC11576871; doi:10.1007/s11096-024-01781-3)
Supplement: Supplementary file 2 — Supplementary file2 (DOCX 26 KB) [file 11096_2024_1781_MOESM2_ESM.docx]

Table 2 Adapted “Patient Care and Clinical Pharmacy Skills” domain of European Common Training Framework (CTF) for the national hospital clinical pharmacy context of Austria

| **Patient care and clinical pharmacy skills competencies** | | | | | | |
| --- | --- | --- | --- | --- | --- | --- |
| **Competencies** | | **Attitude** | **Knowledge** | | **Behaviour Competencies** | |
| 1. | Patient consultation | Patient oriented and safety attentive, reliable and confident | 1.1 | Patient assessment | 1.1.1 | Can take a health status and medication history directly from the patient where this is possible, or using medical records or other relevant information sources as appropriate, to identify conditions, symptoms and the specific needs of individual patients. |
|  |  |  |  |  | 1.1.2 | Can retrieve all appropriate, relevant and available information about patients’ health, social status and ethnic background from different sources. |
|  |  |  |  |  | 1.1.3 | Knows how basic physical health examinations are performed (for example: blood pressure, body mass index), can interpret and respond appropriately to such data, and when required can carry out relevant components of basic physical examination. |
|  |  |  |  |  | 1.1.4 | Can document an accurate and comprehensive medication history (including medical devices, if appropriate), including information about adherence, adverse effects, and outcomes. |
|  |  |  |  |  | 1.1.5 | Discuss and agree with patients the appropriate use of medicines, taking into account patients’ preferences. |
|  |  |  | 1.2 | Patient consent  (if applicable) | 1.2.1 | Ensures that patients receive appropriate and sufficient information to obtain informed consent when this is required for procedures, treatment or research. |
|  |  |  | 1.3 | Consultation or referral | 1.3.1 | Can appropriately refer, or seek advice about complex pharmaceutical and/or complex healthcare issues from a senior or specialist colleague, or other healthcare professional, or other service. |
|  |  |  |  |  | 1.3.2 | Ensures the judicious use of standard operating procedures and guidelines, especially for complex situations. |
|  |  |  |  |  | 1.3.3 | Advises patients when and what circumstances in which to seek further medical intervention. |
|  |  |  |  |  | 1.3.4 | Identifies opportunities to engage in health promotion. |
| 2. | Medicines, medication safety and medical devices | Safety attentive, reliable and confident | 2.1 | Ensure appropriate selection of dosing regimen: formulation and concentration, route and timing. | 2.1.1 | In collaboration with other relevant health care professionals, ensures the right medicine is given for the right reason, in the right dose, using the right route of administration, at the right time, with the right documentation, to the right person. |
|  |  |  |  |  | 2.1.2 | Understands the attributes of the full range of formulations available, and routes of administration, to ensure optimal choice of medication. |
|  |  |  |  |  | 2.1.3 | Considers whether medical devices for administration are required to ensure safe and effective administration. |
|  |  |  |  |  | 2.1.4 | Ensures optimal time of dose has been chosen and provides appropriate solutions for missed, delayed and duplicate doses. |
|  |  |  | 2.2 | Medication therapy management | 2.2.1 | Can evaluate patient-specific drug therapy and therapeutic problems, through monitoring of patients in health care settings. Achieved by evaluation of disease progression, any disease related complications, efficacy of drug therapy, and any drug-related adverse effects. |
|  |  |  |  |  | 2.2.2 | Can design a comprehensive drug therapy plan for patient specific problems. |
|  |  |  | 2.3 | Medication reconciliation | 2.3.1 | Uses an accurate and comprehensive medication history, including the use of medical devices, to identify any discrepancies and reconcile medicines, in collaboration with patients, carers and other relevant health care professionals. |
|  |  |  |  |  | 2.3.2 | Communicate and resolve any identified medicines discrepancies with patients, prescribers, community pharmacist, nurse, or patient carer. |
|  |  |  |  |  | 2.3.3 | Document all reconciliation changes in the appropriate records. |
|  |  |  | 2.4 | Pharmacoeconomics | 2.4.1 | Can identify cost effective medicines and medical devices using valid and relevant pharmacoeconomic data. |
|  |  |  | 2.5 | Medication safety | 2.5.1 | Discusses medication safety issues with other staff, identifies hazardous practices, contributes to the implementation of new procedures and practices to deal with medication safety risks or issues. |
|  |  |  | 2.6 | Medicines management problems | 2.6.1 | Can identify patients for which ongoing monitoring is required together with other responsible healthcare professionals. This includes monitoring parameters. |
|  |  |  |  |  | 2.6.2 | Can establish a plan for review of objectives and treatment outcomes together with other responsible healthcare professionals. |
|  |  |  |  |  | 2.6.3 | Can ensure appropriate action is taken promptly when medicines management issues area identified and ensures that the required actions are implemented, to ensure that patient harm is avoided or minimised. |
|  |  |  | 2.7 | Seamless care  (* focus on medicine management) | 2.7.1 | Identifies and manages medicines-management and transfer of pharmaceutical care related to the healthcare interface. |
|  |  |  |  |  | 2.7.2 | Identifies and manages the problems related to switch patient’s medication to formulary medicines especially in specific groups like elderly and paediatric patients. |
|  |  |  | 2.8 | Transfer of care  (* focus on communication) | 2.8.1 | Communicates with other organisations that affect the delivery of patient care, especially involving the transfer of care, and including information on reconciliation of medication on the base of the patient’s acute state and specific needs. |
|  |  |  | 2.9 | Multidisciplinary team | 2.9.1 | Pharmacy team members are integrated into multidisciplinary teams across the organisation and provide patient facing clinical services to ensure safe and appropriate medicines use for all patients, whatever the setting. |
|  |  |  |  |  | 2.9.2 | Patients, medical and nursing teams have access to pharmacy expertise when needed. Specialist/advanced/consultant level pharmacists work in clinical specialties to maximise the availability of expert resource to other members of the multidisciplinary team for the benefit of patients receiving care in that area. |
|  |  |  |  |  | 2.9.3 | As part of a multidisciplinary team, the pharmacy team monitor patients’ responses to their medicines. Appropriate action is taken where problems (potential and actual) are identified. |
| 3. | Information and communication | Critically thinking while gathering the information | 3.1 | Accessing and summarising medicines related information | 3.1.1 | Can effectively find and identify relevant and valid information using specific pharmacy expert databases, information services, evidence-based literature and relevant local summaries of evidence to promote rational and safe use of medicines. |
|  |  |  |  |  | 3.1.2 | Can use medicines information effectively in order to undertake a review and provide a summary of the rational and safe use of medicines. |
|  |  |  |  |  | 3.1.3 | Can apply relevant and valid evidence-based data for medicine and medical devices use to the care of individual patients. |
|  |  |  |  |  | 3.1.4 | Provides medicines information in response to queries in a manner appropriate to the recipient. |
|  |  |  | 3.2 | Appraisal options | 3.2.1 | Assesses options available for problem solving, considering possible outcomes of any actions. |
|  |  |  | 3.3 | Decision making and logical approaches to decision making | 3.3.1 | Understands and demonstrates clear decision making actions. Can identify the most appropriate decision pathways. |
|  |  |  |  |  | 3.3.2 | Is aware of one’s own limits and seeks advice when necessary. Knowing what to do when one does not know what to do. |
|  |  |  | 3.4 | Questioning technique | 3.4.1 | Uses appropriate communication and questioning techniques to gather relevant patient information. |
|  |  |  |  |  | 3.4.2 | Determine who the most appropriate person is to discuss the patient’s medicines with. |
|  |  |  | 3.5 | Documentation (intervention) | 3.5.1 | Documents interventions and maintains appropriate records. |
|  |  |  |  |  | 3.5.2 | Accurately and succinctly document the nature of the intervention in the patient’s health record and/or medication management plan according to local policy. |
|  |  |  | 3.6 | Documentation (medication incidents) | 3.6.1 | Initiate reporting of medicines-related events or circumstances which could have, or did lead to unintended harm to a person, loss or damage, and/or a complaint, according to local policy. |
| 4. | Providing information | Helpful and good communicator | 4.1 | Provides accurate information | 4.1.1 | Ensures information provided is accurate, validated and understandable to other professionals, patients and carers, according to their needs. |
|  |  |  |  |  | 4.1.2 | Uses effective verbal, non-verbal, listening and written communication skills to communicate clearly, precisely and appropriately. |
| 5. | Expert practice | Responsible for own lifelong learning and development | 5.1 | Pathophysiology and anatomy | 5.1.1 | Understands normal organ anatomy and function, the effects of disease states that affect medicines use. |
|  |  |  |  |  | 5.1.2 | Can apply knowledge of pathophysiology to specific therapeutic areas and to particular patient groups (e.g. paediatric, older people, etc). |
|  |  |  | 5.2 | Pharmacology and Pharmacotherapy | 5.2.1 | Can describe and discuss the pharmacology and pharmacotherapy of drugs in routine use. |
|  |  |  |  |  | 5.2.2 | Is aware of and can discuss the importance of emerging technologies in pharmacology and pharmacotherapy (e.g. pharmacogenomics). |
|  |  |  | 5.3 | Pharmaceutical technology & biopharmacy | 5.3.1 | Understands the scientific basis of different dosage formulations, how they are designed and how they may influence the clinical efficacy of medicines. |
|  |  |  |  |  | 5.3.2 | Understands how administration, drug distribution, drug elimination influences medicines outcomes (ADME, pharmacokinetics, biopharmacy). |
|  |  |  |  |  | 5.3.3 | Is able to understand the advantages and risks of the new formulations (e.g. nanotechnology, Advanced Therapy Medicinal Products [ATMPs]). |
|  |  |  |  |  | 5.3.4 | Has knowledge on the purposes and risks of the adjuvant compounds in medicines formulations. |
|  |  |  |  |  | 5.3.5 | Is able to make a patient oriented choice for the best individual pharmaceutical formulation to obtain optimal outcomes. |
|  |  |  | 5.4 | Adverse effects and toxicology | 5.4.1 | Can recognise side effects of medicines and, in collaboration with other health care professionals, patients and carers, can recommend alternative treatment strategies and is able to take action, when required. |
|  |  |  |  |  | 5.4.2 | Can support the management of acute toxicity and advise on appropriate antidotes. |
|  |  |  |  |  | 5.4.3 | Contributes to a national reporting system of pharmacovigilance, identifying, recording and reporting suspected or confirmed adverse drug reactions, sensitivities or allergies. |
|  |  |  | 5.5 | Microbiology and hygiene | 5.5.1 | Can describe the microbiological interactions associated with pharmacotherapy including resistance mechanisms and anti-microbial stewardship. |
|  |  |  |  |  | 5.5.2 | Can support staff training activities regarding infection control and infection control strategies. |
|  |  |  | 5.6 | High-alert medications | 5.6.1 | Can identify high-risk medicines and high-risk administration of medicines relevant to the healthcare setting. |
|  |  |  |  |  | 5.6.2 | Can plan and implement medicines management actions to minimize the risk related to these medicines. |
|  |  |  |  |  | 5.6.3 | Assesses the potential for inappropriate use, misuse or abuse of medicinal treatments. |
|  |  |  |  |  | 5.6.4 | Identification of patients most at risk of medication misadventure 🡪 Identify if patient is at risk of medication misadventure. |
|  |  |  | 5.7 | Medical devices | 5.7.1 | Understands and applies knowledge of medical devices for drug delivery, administration and reconstitution. |
|  |  |  | 5.8 | Research | 5.8.1 | Can describe, explain and discuss commonly used research methodologies in order to be able to participate in pharmacy practice research and clinical research. |
|  |  |  |  |  | 5.8.2 | Can use benefit-risk assessments for evaluating alternative treatment strategies. |
|  |  |  |  |  | 5.8.3 | Can plan, lead on, and conduct research/practice development projects* to promote safe and rational use of medicines. Able to collaborate with other healthcare professionals. * this includes audit and service evaluation projects. |
|  |  |  | 5.9 | Pharmacist prescribing (within the legal authorisations in Austria) | 5.9.1 | Pharmacist prescribers are integrated into relevant care pathways and prescribing regularly. |
| 6. | Other |  | 6.1 | First Aid | 6.1.1 | Applies first aid when required and acts to arrange follow-up care. |
|  |  |  | 6.2 | Logistics (in specialised settings) | 6.2.1 | Ensure patients’ medicines are available from the time that their next dose is needed minimising missed doses of medicines. |
|  |  |  | 6.3 | Ethics | 6.3.1 | Respects the rights of patients in therapeutic decisions and assists in providing information to facilitate their decision (e.g. informing patients on risks associated with medicines). |
|  |  |  | 6.4 | Record of contributions (if applicable) | 6.4.1 | Documents information to support contributions to patient care, for example maintains pharmaceutical care plans. |
